# Ambiguity Detection in Medical Exams via Large Language Models: Retrospective Cross-Sectional Pilot Study

**DOI:** 10.2196/82702

**Published:** 2026-05-26

**Authors:** Romain Lombardi, Alexandre Destere, Jean Dellamonica, Alexandre O Gérard, Mathieu Jozwiak

**Affiliations:** 1 Critical Care Unit, Pasteur 2 University Hospital, 30 Voie Romaine, Nice, 06100, France, 33 0669032616; 2 UR2CA, Unité de Recherche Clinique Côte d'Azur, Université Côte d'Azur, Nice, France; 3 Department of Clinical Pharmacology and Pharmacovigilance Center, Medical Centre, Université Côte d'Azur, Nice, France; 4 Maasai Team, Laboratoire J.A. Dieudonné, Université Côte d'Azur, Inria, CNRS, Nice, France; 5 Critical Care Unit, Archet 1 University Hospital, Nice, France

**Keywords:** large language model, LLM, medical education, exams, ambiguity detection, automated scoring, quality assessment, docimology, artificial intelligence, critical care, emergency

## Abstract

**Background:**

Large language models (LLMs) have emerged as promising tools in medical education due to their ability to understand, generate, and reason with natural language. Their ability to simulate expert reasoning suggests a potential for supporting quality control in assessment design. In this study, the use of LLMs in identifying ambiguous or poorly constructed exam items in critical care academic assessments was evaluated.

**Objective:**

The study aimed to develop automated ambiguity and quality scores to objectively assess individual questions and entire exam components.

**Methods:**

We analyzed 264 questions from academic exams conducted over 3 academic years (2023-2025) at the Medical School of Université Côte d’Azur. Questions were drawn from 4 docimological formats: progressive clinical cases (PCC), mini-PCC, key feature problems, and isolated question sequences (IQS). Each element was submitted to 4 LLMs (ChatGPT, Gemini Pro, Le Chat, and DeepSeek) without prompt engineering. Performance was evaluated using the official correction key. We applied 4 binary diagnostic tags based on model agreement and self-reported ambiguity: ambiguity, low performance, incoherence, and subjective ambiguity. These tags generated a composite ambiguity score and contributed to a weighted quality score for each exam component.

**Results:**

LLMs achieved mean scores in the same range as students, with no significant differences across academic years and significantly higher performance on the mini-PCC and IQS formats (*P*=.049 and *P*=.04, respectively). IQS items had the highest ambiguity scores (54 items received a score of 2 in both 2023 and 2024, and 53 items retained the same score). Tag patterns revealed frequent issues with ambiguity and inconsistency. Quality scores varied across academic years. IQS predominantly showed moderate ambiguity (score 2), with occasional instances of strong signals. There was no significant difference in quality based on author specialty or seniority (*P*=.08 and *P*=.44, respectively).

**Conclusions:**

In this pilot study, LLMs may offer a preliminary framework to proactively detect ambiguous exam questions and estimate the overall quality of an exam. Integrating these tools into the assessment design process could potentially reduce the need for postexam corrections and may help improve fairness and clarity in medical evaluations.

## Introduction

Large language models (LLMs) are a class of artificial intelligence (AI) systems based on neural network architectures designed for natural language understanding and generation [1]. To function efficiently and deliver robust performance, LLMs are trained on massive amounts of textual data. They are designed to identify and learn recurrent patterns embedded in natural language [2]. LLMs have become an integral part of daily applications. Chatbot platforms such as ChatGPT, Google Gemini, DeepSeek, and Mistral are leading examples of the widespread use of natural language interactions [3-7]. LLMs are also extensively applied in machine translation, with systems like DeepL and Google Translate providing accurate and context-aware translations [8]. Furthermore, these models play a growing role in education by generating automated exercises (eg, Socratic) and in software development by providing intelligent code assistance (eg, GitHub Copilot) [9].

LLMs have already been studied in a wide range of medical applications across various disciplines, and they appear to have a promising future [1,2]. Numerous studies in the fields of intensive care and perioperative medicine have investigated the various applications of LLMs. For example, Chung et al [10] demonstrated how these models can be used for perioperative risk stratification. These models can also be used to generate databases from the daily unstructured medical data in the medical sheets of critically ill patients [11]. LLMs can also serve as valuable tools for triaging patients and prioritizing those requiring emergency department care based on severity [12]. In more unconventional applications, their use has been explored for generating recommendations on the most relevant clinical topics. However, the results remain mixed and require expert oversight [13]. Their increasing use in clinical reasoning necessitates both evaluation and rigorous testing to ensure their validity.

LLMs are increasingly being explored as important tools in the fields of pedagogy and medical education [14,15]. These models can be used to train anesthesia residents by generating relevant information and synthesizing content from multiple sources [16]. They can assist with clinical diagnosis, which is particularly valuable for students in the early stages of their medical education [17]. One of the major limitations of clinical examination simulation is the limited availability of standardized patients. ChatGPT may offer a potential solution by serving as a substitute for human actors [18]. AI could enhance not only medical students’ learning but also the evaluation and quality assurance of medical assessments. Maitland et al [19] attempted to have LLMs sit the Membership of the Royal Colleges of Physicians written examinations in the United Kingdom. The results showed that LLMs outperformed human candidates. The fine-tuned models also enabled the identification of 8 types of errors, such as factual errors, context errors, and omission errors. This offers a potential way to improve the quality of exam questions. In a preliminary study, Gérard et al [20] evaluated local and European pharmacology exams, highlighting how LLMs can help in identifying ambiguous questions.

The primary objective of this study was to develop a methodology for detecting ambiguous or low-quality questions in academic exams using various LLMs.

## Methods

### Objectives

We used LLMs as expert reasoning agents to simulate medical students’ behavior and identify exam questions that might be poorly constructed or ambiguous. The secondary objectives were to develop an ambiguity scoring system and a quality score at the case level (ie, aggregating multiple questions), and to analyze quality variations according to academic year, assessment format, and the author’s academic title.

### Data Sources and Study Population

We analyzed academic exams taken by medical students at the Université Côte d’Azur. Only questions from the “Emergency and Intensive Care” course unit were included in this study. The questions were written either by an intensivist or an emergency physician, and the author was either a clinical fellow, an associate professor, or a full professor. A total of 3 academic exam cohorts were analyzed: 2023, 2024, and 2025. An exam was composed of several docimological components, and each component consisted of multiple questions, such as multiple-choice questions (MCQs), short-answer questions (SAQs), extended matching questions, or single-best-answer questions. The docimological components consist of four types: (1) progressive clinical cases (PCC; extended clinical scenarios with sequenced MCQs), (2) key feature problem (KFP; short critical decision-making vignettes), (3) mini-PCC (mPCC; condensed clinical cases), and (4) isolated question sequence (IQS; standalone items [MCQs or SAQs]) [21].

The year 2025 corresponded to the evaluation of master’s level 2 (M2) students, while the years 2024 and 2023 corresponded to evaluations of both master’s level 1 (M1) and M2 students. Additionally, the 2023 cohort also included evaluations of master’s level 3 (M3) students. Each docimological component varied across the academic years.

Each exam consisted of between 3 and 8 different docimological components. A total of 264 questions were analyzed. All the exam scores were normalized to a 20-point scale. Partial credit was awarded based on the number of discrepancies between the participant’s response and the official answer key, specifically false positives or incorrect options omitted. Participants received a score of 0.5 for 1 discrepancy, 0.2 for 2 discrepancies, and no points if more than 2 discrepancies were identified. A minimum score of 10 out of 20 was required to pass the exam.

The official correction and scoring rubric, the global performance scores of students per question and per docimological component, the responses and performance scores from the LLMs, and manual annotations from a human investigator were available for all questions.

To ensure consistency and minimize bias, all questions were carefully standardized in their format and presentation. No alterations or prompt engineering were applied to either the questions or answer options, allowing for an impartial assessment of the LLMs’ inherent capabilities. The exam questions, extracted from PDF files, were submitted to each LLM, which was asked to choose the appropriate answer relying solely on its prior knowledge and training.

### LLMs Evaluation Procedure and Ambiguity Detection

We analyzed the results of the 4 most prominent LLMs: ChatGPT-4, Gemini 2.5 Pro, Mistral Le Chat, and DeepSeek R1. Each exam question was submitted to the LLMs in two formats: (1) full-context input, simulating access to the complete clinical case; and (2) sequential-input, where questions were revealed one by one, mimicking an exam scenario. We deliberately adopted a zero-shot prompting strategy without task-specific parameter tuning or extensive prompt engineering. The rationale was to simulate a real-world usage scenario where educators or examiners interact with LLMs as “out-of-the-box” tools without requiring specialized expertise in AI engineering, and to assess how the intrinsic phrasing of each test item naturally triggers consistency or divergence across different model architectures, minimizing the risk of over-tuning the models.

LLM responses were graded manually by the investigator according to the official correction, using the same scale applied to student answers. We also asked each LLM to identify any ambiguity in the question based on its own defined criteria. From these scores, three metrics were computed per question: the mean score of the LLMs, the SD of the LLMs’ scores, and the number of LLMs that failed to answer correctly. Of note, no significant differences were observed between the 2 input formats.

We implemented a tagging system to assess the quality and clarity of each question. This system assigned binary diagnostic tags based on LLM performance patterns. These tags were designed to identify specific aspects of flawed question design and were applied automatically to each question to minimize the impact of subjective human bias. The tags used are as follows:

- Ambiguity tag: assigned when multiple LLMs provide incorrect answers with a diversity of distinct wrong responses, indicating disagreement in correctness, reasoning, or interpretation of the question. The tag was set to 1 if at least 2 LLMs answered incorrectly with at least 2 different incorrect answers among them.
- Low performance tag: this tag indicates that the question is challenging or potentially flawed due to universally poor performance by the LLMs. The tag is assigned if the mean normalized score of all LLMs is below 0.5, combined with low variance among their scores (SD<0.3). The low performance tag aims to identify items that are potentially “unanswerable” or structurally flawed. While difficulty and ambiguity are distinct, a universal failure of multiple expert-level LLMs with low variance often points toward a lack of clarity or a misleading structure rather than intended pedagogical selectivity.
- Incoherence tag: captured inconsistency in LLMs’ understanding of the question. The tag was assigned when the measure of dispersion in LLM scores exceeded 0.3 (SD>0.3).
- Subjective ambiguity detection is assigned when the LLMs themselves identify ambiguity in the question based on evaluation criteria that they have autonomously established. The tag was set to 1 if at least 2 LLMs reported the presence of ambiguity according to their self-defined standards.

Table 1 summarizes the different tags and their assignment criteria.

**Table 1.** Summary of definitions and criteria for tag attribution.

Tag names	Definition	Operational criteria	Rationale
Ambiguity tag	Multiple LLMs^a^ provide incorrect answers with a diversity of wrong responses.	Tag=1 if at least 2 LLMs answer incorrectly and if at least 2 different answers	Reducing false positives arising from multiple LLMs converging on the same wrong answer reflects a shared misconception rather than true ambiguity. The diversity of wrong answers suggests multiple plausible interpretations or poorly defined answer keys.
Low performance tag	The question is challenging or potentially flawed due to universally poor performance by the LLMs	Tag=1 if the mean normalized score of all LLMs is below 0.5 and SD<0.3	Combined criterion distinguished truly difficult but valid questions from ambiguous or flawed questions (displaying greater variability or discrepancy between LLMs and student results).
Incoherence tag	Captures inconsistency in LLMs’ understanding of the question	Tag=1 if SD>0.3	Prevents outliers from skewing variability measures and ensures that incoherence reflects genuine divergence in interpretation rather than isolated anomalies.
Subjective ambiguity detection tag	Assigned when LLMs identify ambiguity based on their own evaluation criteria	Tag=1 if at least 2 LLMs report the presence of ambiguity	This approach leverages the LLMs’ capacity for self-assessment. Requiring agreement from multiple models helps ensure that the detected ambiguity is not idiosyncratic to a single LLM.

a LLM: large language model.

Each question was automatically tagged with the 4 binary flags described earlier. The sum of these tags formed a composite ambiguity score ranging from 0 to 3 (ambiguity score = ambiguity tag + low performance tag + incoherence tag + subjective ambiguity detection tag).

A score of 0 indicated “No issues detected,” a score of 1 indicated “Minor concern,” a score of 2 indicated “Moderate ambiguity,” and a score of 4 indicated “Strong signal of item flaw or misleading structure.” The low performance and incoherence tags are mutually exclusive, as they depend on opposing SD thresholds.

The thresholds for flagging potentially problematic questions were systematically determined using a combination of visual inspection and data-driven analysis, specifically elbow-point detection, to ensure that tag assignments were objectively grounded in the performance distribution. The elbow-point detection technique identifies the inflection point in the sorted distribution of each metric, representing the transition from typical to anomalous values.

### Quality Assessment for Docimological Component

To calculate the quality score for each docimological component, we first determined the average score obtained by the LLMs for each question. We then applied a weighting based on the ambiguity score of each question according to the following formula:


(1) $$Q_{i}\text{=}20\times S_{i}$$

where *Q_i_* is the quality score for question *i* (on a 20-point scale), and *S_i_*, ranging from 0 to 1, is the average score of LLMs for question *i*.


(2) $$Score_{component}\text{=}\frac{\sum_{i\text{=}1}^{N}Q_{i}\times w_{i}}{\sum_{i\text{=}1}^{N}w_{i}}$$

where N is the total number of questions, *Q_i_* is the quality score for question *i* (on a 20-point scale), and *w_i_* is the weight assigned to question *i* with:


(3) $$w_{i}\text{=}\frac{1}{1\text{+}A_{i}}$$

where *w_i_* is the weight assigned to question *i*, and *A_i_* is the ambiguity score for the question *i*.

Using this system, we were able to assign a qualitative description to each docimological component based on the score obtained, as follows:

- If score ∈ [18-20]: excellent, clear, precise, and unambiguous writing.
- If score ∈ [15-18]: very good, well-structured writing with minor ambiguities.
- If score ∈ [12-15]: moderate, understandable writing, but with recurring ambiguities.
- If score ∈ [10-12]: poor, confused writing with repeated ambiguities.
- If score ∈ [0-10]: insufficient, vague, and ambiguous writing impacts reliability.

Components with a quality score below 15 were re-evaluated by the authors to clarify the identified ambiguities.

### Statistical Analysis

The results obtained by the students were expressed in terms of mean, median, and maximum scores, whereas the LLMs’ results were presented as mean only. We used the Mann-Whitney *U* test to compare the performance of LLMs and students, with the results expressed in terms of *P* values. We compared the quality score results according to the writer’s medical specialty, professional career level (clinical fellow, associate professor, or full professor), and the type of docimological component. A Student 2-tailed *t* test was used when the distribution was normal, as assessed by the Shapiro-Wilk test; otherwise, a Mann-Whitney *U* test was applied. We considered a *P* value less than .05 to be statistically significant.

### Ethical Considerations

All materials analyzed were anonymized. No individual student responses were accessed. LLMs were used only to simulate reasoning without personalization. According to French law (Articles L1121-1 et seq. of the French Public Health Code) [22], this study did not qualify as biomedical research involving human participants. Since it involved neither interventions on individuals nor the processing of identifiable personal data, it was exempt from ethics committee review. Consequently, under French regulations [23], this type of research did not require approval from an institutional ethics board.

### Development Environment

All the analyses and development were performed in Python (Python Software Foundation) version 3.13.3, along with the following libraries and their respective versions: *pandas* 2.2.3, *NumPy* 2.2.6, *seaborn* 0.13.2, *matplotlib* 3.10.3, *scikit-learn* 1.6.1, *statsmodels* 0.14.4, *SciPy* 1.15.3, and *UpSetPlot* 0.9.0.

## Results

### Performance Comparison

A total of 264 exam questions were analyzed and submitted to the 4 LLM models (ChatGPT, Gemini Pro, Le Chat, and DeepSeek). Students achieved relatively consistent results over the years, with average scores ranging from 11.59 (SD 2.18) to 14.0 (SD 1.80) (Table 2). Performance was higher on KFPs for students. The results obtained by LLMs across academic years were heterogeneous. Superior performance was achieved by ChatGPT and Gemini Pro. No significant difference was observed between the LLMs’ results and the students’ results when comparing across academic years. However, when analyzing performance by docimological component, the LLMs outperformed students in the mPCC and IQS formats (*P*=.049 and *P*=.04; respectively).

**Table 2.** Performance comparison between large language models (LLMs) and students’ scores. For the LLMs, only the mean score is reported.

	Students			LLM model				*P* value
	Median (IQR)	Mean (SD)	Min-max	ChatGPT	Gemini Pro	Le Chat	DeepSeek	
Academic level								
2023								
M1^a^	11.85 (10.09-13.15)	11.59 (2.18)	5.35-16.55	15.28	18.44	12.28	11.8	.05
M2^b^	12.80 (11.68-13.71)	12.64 (1.48)	7.39-18.42	14.11	16.77	12.32	12.27	.39
M3^c^	12.53 (11.62-14.03)	11.77 (4.19)	20 (0-20)	—^d^	—^d^	—^d^	—^d^	.40
2024								
M1	14.14 (12.87-15.25)	14.0 (1.80)	7.54-15.82	15.91	14.36	15.75	14.30	.17
M2	13.07 (11.96-14.15)	13.08 (1.63)	8.40-17.36	12.88	16.30	13.21	12.52	.61
2025								
M2	11.68 (10.61-12.74)	11.67 (1.56)	7.35-16.46	11.32	12.38	11.79	11.96	.74
Docimological component								
PCC^e^	12.67 (10.18-14.86)	12.07 (4.49)	0.0-20	13.32	16.40	13.10	11.81	.16
mPCC^f^	11.85 (8.35-15.0)	11.35 (5.26)	0.0-20	13.16	15.14	13.00	12.78	.049
KFP^g^	13.33 (8.0-15.33)	11.91 (5.71)	0.0-20	14.95	14.19	14.95	14.19	.16
IQS^h^	10.14 (9.3-13.40)	10.63 (4.15)	0.0-18.4	14.14	16.22	12.71	12.49	.04

a M1: master’s level 1.

b M2: master’s level 2.

c M3: master’s level 3.

d The exam cases provided to M2 and M3 students during faculty examinations are strictly identical.

e PCC: progressive clinical cases.

f mPCC: mini–progressive clinical cases.

g KFP: key feature problem.

h IQS: isolated question sequence.

### Ambiguity Score and Tag Repartition

**Figure 1.**
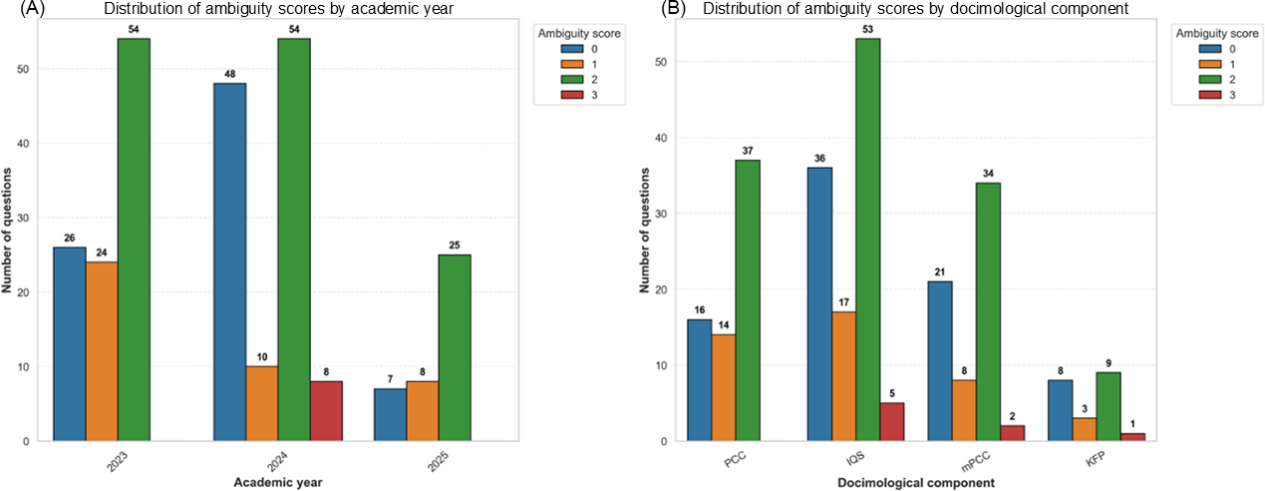
Distribution of ambiguity scores by item according to (A) academic year and (B) docimological component. No scores greater than 3 are shown, as the tagging criteria make this situation impossible. IQS: isolated question sequence*;* KFP: key feature problem*;* mPCC: mini–progressive clinical cases; PCC: progressive clinical cases.

Figure 1 shows the distribution of ambiguity scores by item according to academic year and docimological component. An ambiguity score of 2 was the most frequently observed, regardless of academic year or type of docimological component. Specifically, 54 items received a score of 2 in both 2023 and 2024, while 53 items had the same score in the IQS category, and 37 and 34 items in the PCC and mPCC categories, respectively. The 2024 academic year had the highest number of ambiguity scores at 0, and there was no score greater than 2 in 2023 and 2025. IQSs exhibited the highest number of scores above 2, with a total of 5 items scoring 3, but also the highest number of scores at 0, with 36 questions in total.

**Figure 2.**
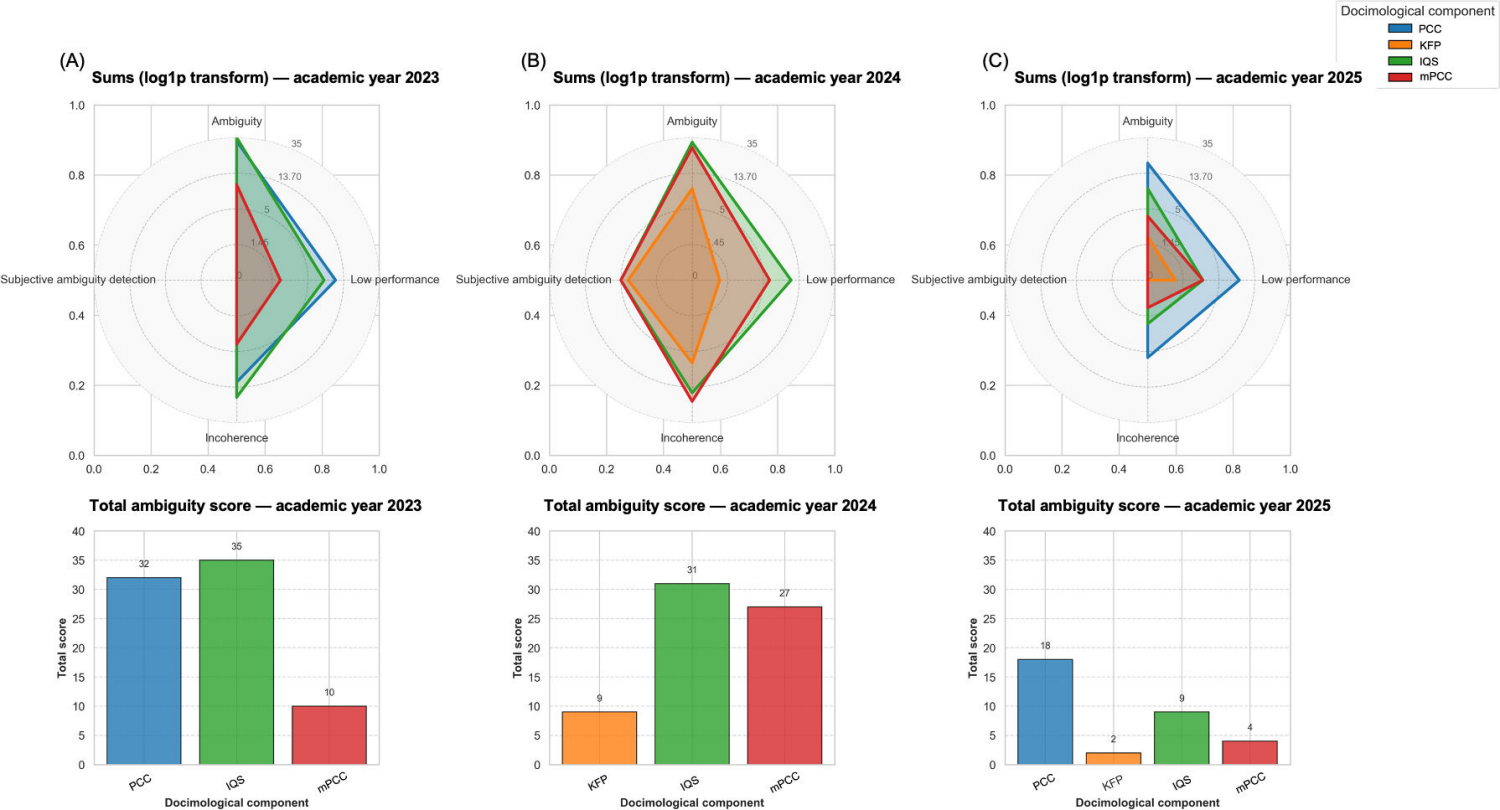
Tag repartition by docimological component according to (A) academic year 2023, (B) academic year 2024, and (C) academic year 2025. To increase the visibility of the differences between the distributions of the different tags, the axes of the radar plots were transformed using log1p. The total ambiguity score results from the sum of all the ambiguity scores. IQS: isolated question sequence*;* KFP: key feature problem*;* mPCC: mini–progressive clinical cases; PCC: progressive clinical cases.

For the academic year 2023, the low-performance and ambiguity tags were over-represented compared to the tags inconsistency and subjective ambiguity detection, regardless of the docimological component studied (Figure 2). The total ambiguity score was higher for IQS and PCC, with scores of 35 and 32, respectively. An increase in the number of subjective ambiguity detection tags was observed in 2024, while the distribution of the other tags remained unchanged. During that year, the total ambiguity score for mPCC increased to 27, compared to 10 in 2023. No PCC case was included that year. KFPs had the lowest total ambiguity score during the same period. In 2025, the various tags appeared less frequently than in previous years. The subjective ambiguity detection tag was not used, and the maximum total ambiguity score for the PCC cases was 18.

Figure S1 in Multimedia Appendix 1 shows the most common tag combinations. The most frequent tag combinations were ambiguity and incoherence (78, 29.5% items), low performance and ambiguity (52, 19.7% items), and incoherence, subjective ambiguity detection, and ambiguity (5, 1.9% items). The ambiguity tag alone appeared in 36 items, corresponding to 13.6% of cases, and the low performance tag was never found alone.

### Quality Score

**Figure 3.**
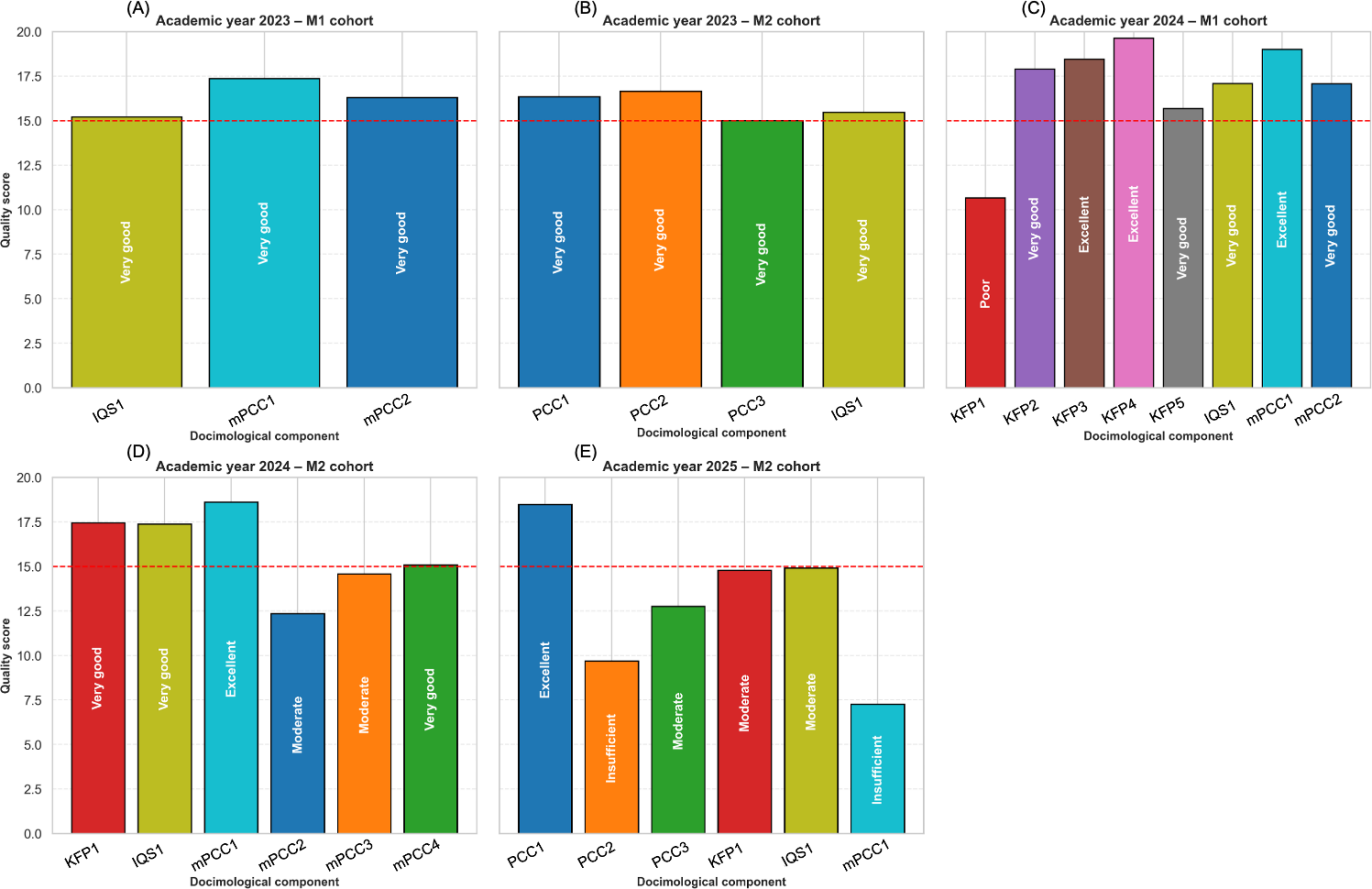
Qualitative score distribution by academic year and cohort. (A) 2023—M1 cohort, (B) 2023—M2 cohort, (C) 2024—M1 cohort, (D) 2024—M2 cohort, and (E) 2025—M2 cohort. IQS: isolated question sequence*;* KFP: key feature problem*;* mPCC: mini–progressive clinical cases; PCC: progressive clinical cases.

All the docimological elements for the faculty exams in 2023 exceeded the validity threshold, with a qualitative score of 15 out of 20, and all of them received a “Very good” rating (Figure 3). For the 2024 M1 cohort, only 1 component, KFP1, fell below the validity threshold with a qualitative rating of “Poor,” whereas for the M2 cohort, 2 components were below this threshold with ratings of “Moderate.” Four of 6 items did not reach the 15 out of 20 threshold for the 2025 M2 cohort, with the lowest scores observed for PCC2 and mPCC1, both receiving a qualitative rating of “Insufficient.” Figure 3 also shows an expansion in the diversity of docimological element types over the years, with all formats (PCC, mPCC, KFP, and IQS) represented in 2025. In 2023, and for the M1 academic exam, only the IQS and mPCC formats were included. IQSs were represented in each academic exam. Elements with a score of 15 were reviewed by the authors, who confirmed the presence of ambiguities in the wording, structure, or context.

No significant differences were found between the groups when comparing results based on quality scores according to the writer’s specialty and career level, nor when comparing results by career level and the docimological component (Table S1 in Multimedia Appendix 2). No significant difference in career level was found between clinical fellows and associate professors (*P*=.44). The difference between emergency writers and intensive care unit or critical care unit writers appeared more pronounced, approaching statistical significance (*P*=.08). After adjustment for career level, the quality gap between mPCC and KFPs remained more marked than for the remaining docimological types (*P*=.12 and *P*=.16, respectively). The number of contributors involved in writing the exams increased during 2025, particularly with more clinical fellows participating.

## Discussion

### Principal Findings

In our comparison of LLMs with average student scores, no significant differences were found across academic years. However, mean scores varied between LLMs, and additional differences appeared when analyzing performance by type of docimological component. One reason for this variation may be the specific training approach and intended purpose of each model. ChatGPT and Gemini Pro are the oldest and most widely known models. A study focusing on the responses of various LLMs to medical questions related to tuberculosis highlighted the absence of overall performance differences between the models but revealed disparities depending on the type of content submitted [24]. It is important to emphasize that this study was not designed to establish the superiority of 1 specific LLM over others. Rather, building upon the methodological framework previously described by our team in Gérard et al [20], our approach focuses on the synergy of an ensemble of models. Using multiple LLMs allows for a convergence of evidence, which increases the reliability of ambiguity detection by filtering out idiosyncratic errors or “hallucinations” from individual models.

There was an unequal distribution of tag types and ambiguity scores depending on the academic year and the type of assessment elements. The highest ambiguity score was observed for IQSs. One potential explanation for the disparity in distribution across academic years, though not statistically confirmed, might be differences in authorship. In 2024 and 2025, a greater number of contributors, particularly clinical fellows, were involved in writing the exams. While these younger authors are often tasked with drafting clinical case scenarios, our analysis did not reach statistical significance to definitively confirm a difference in quality based on academic rank. This lack of significance is likely due to a lack of statistical power when subdividing the 264 items by year, format, and seniority. This idea is supported by several studies. For example, Oyibo et al [25] examined a cohort of 65 junior doctors and found that young professionals produced case scenarios that were rated as effective and clear in 98.5% of cases. Additionally, they were enthusiastic about the writing process. Furthermore, case writing is an effective way to develop advanced writing skills, which are particularly useful for creating clinical case scenarios [26]. However, in our study, we did not find a statistically significant difference in the quality of clinical cases according to professional grade or specialty. Another explanation can be found in the type of docimological component. The construction of decontextualized single-question items is challenging and leads to a lack of validity and low rigor. Several authors describe IQSs as poor evaluators that encourage memorization rather than critical thinking, making them less discriminative [27,28].

Our results suggest that quality scores could provide a valuable framework for evaluating the ambiguity and overall quality of academic exam cases *a priori*. Unlike current methods that assess items *a posteriori* based on student performance, our approach aims to identify “structural fragility” before the exam is administered. Most evaluations of these components are currently conducted retrospectively, after the exam has been administered [29]. These items may be removed or may affect the exam’s discriminatory power, leading to student frustration. Their removal is costly in terms of time and budget and undermines educators’ credibility and trust among students. Defining a score before submission could provide valuable feedback and may facilitate proactive efforts to improve clarity and revise ambiguous items [30,31].

This work highlights the growing importance of LLMs in the evaluation and validation of academic exams. This study extends the work initiated by Gérard et al [20] in the domain of pharmacological exams by incorporating a rigorous methodology for question evaluation, enabling real-time improvement. In contrast to their findings, our results revealed fewer statistically significant differences between the performance of LLMs and medical students. Our study encompassed a wider array of question formats, including radiological interpretation tasks and docimological components, which were not addressed in the original study. Recent studies have shown that LLMs can do more than just detect ambiguity; they can also analyze complex medical questions, identify nuances in item clarity, and provide consistent evaluative feedback [32,33]. Furthermore, LLMs have been explored as valuable tools for designing and revising educational assessments. They improve item quality and reduce ambiguity through iterative review processes [19]. Our study presented a practical application of LLMs in an understudied field of medical education. LLMs were not used to replace students in taking exams but to offer a promising approach to addressing ambiguities and docimological errors. Our methodology adopts a “human-in-the-loop” approach, where LLMs serve as automated screening tools rather than final judges of construct validity. By flagging questions with low performance or high ambiguity, the system is intended to assist pedagogical experts in concentrating their review on items most likely to contain docimological errors. This synergy between automated detection and human expertise ensures that the distinction between a “challenging but fair” question and an “ambiguous” one is ultimately determined by the educator.

Given the pilot nature of this study and the specific context of critical care assessments, these results should be interpreted with caution. While the trends observed are encouraging, they represent preliminary insights that require validation through larger, multicentric studies across diverse medical disciplines.

### Strengths

This was an original research study in terms of both its thematic focus and its methodological design. We introduced a fully objective tagging methodology aimed at reducing the risk of bias associated with human interpretation. This approach has the potential to be applied beyond the fields of critical care and emergency medicine. The academic exams at the Medical School of Université Côte d’Azur incorporated a variety of docimological formats, including KFP, PCC, mPCC, IQS, MCQ, single-best-answer questions, and SAQ. This study enabled an evaluation of this broad spectrum and an analysis of the various types of errors that may occur during the design of such items. Including this diversity enhances external validity and supports its applicability in real-world educational settings. Comparative analyses with students are often lacking in AI-related studies. Similar to the approach taken by Gérard et al [20], conducting such an analysis provides additional insight into the difficulty level of the assessed questions. This approach allows for a type of *a priori* evaluation, in which the exams are assessed before students take them. This is different from *a posteriori* methods, which rely on student performance data. Thus, this *a priori* evaluation could significantly reduce the logistical and human resources required for postexamination modifications and reviews while supporting fairness among students. To ensure maximum objectivity, we selected the four most widely used LLMs: ChatGPT, Gemini, Le Chat, and DeepSeek. This choice reduced the risk of systematic errors inherent to a single model and was intended to enhance the robustness of the results by converging ambiguity detection across multiple models. Furthermore, no prompt engineering was used to maintain the most realistic and standardized conditions possible.

### Limitations

One of the main limitations of this study was the absence of a systematic human gold standard for the entire dataset. Consequently, we must acknowledge that some ambiguities identified by the LLMs may reflect intrinsic model limitations or reasoning issues rather than actual flaws in the questions. To mitigate this risk of “model noise,” we used an ensemble strategy where ambiguity is only flagged upon the disagreement of multiple independent architectures. Rather than definitive proofs of error, these scores should be viewed as objective indicators of an item’s structural fragility, designed to guide educators toward the questions most in need of manual refinement. Nevertheless, we used four different LLMs to minimize this potential bias. We only used general-purpose models for the analysis of exam questions, although it is known that domain-specific LLMs, such as Med-PaLM 2, exist [34]. These specialized models may demonstrate superior reasoning abilities in complex clinical scenarios and a greater capacity to detect ambiguities in medical questions. However, this would reduce the generalizability of the methodology to domains outside of medicine. Furthermore, the absence of task-specific tuning and the reliance on the generative intuition of LLMs may introduce certain biases. As this study did not use few-shot examples or chain-of-thought prompting to standardize the reasoning process, the “ambiguity scores” should be interpreted as indicators of potential fragility rather than definitive proofs of item flaws. Future research should investigate whether advanced prompting techniques or fine-tuning on human-validated “gold standards” can further isolate model noise from item-specific ambiguity. The monocentric nature of the study population, which is limited to the field of intensive care and emergency medicine, may limit the generalizability of the results. However, the methodology is transferable and can be applied to other specialties. To minimize this bias and avoid focusing on a single student cohort, we included multiple academic years, thereby introducing variation among authors. The use of the “subjective ambiguity” tag may have led to confusion and introduced interpretation bias, as its definition relies on LLMs’ ability to detect ambiguity. This undermines transparency, as the criteria were defined by the models themselves and were not externally verifiable, thereby increasing the risk of a “black box” effect. While our thresholds were derived from data-driven inflection points, we acknowledge that sensitivity analyses across different medical specialties would further validate the stability and generalizability of these assignments. Furthermore, our analysis of quality based on author seniority and specialty may have been limited by a lack of statistical power. Although the total sample of 264 questions is substantial, the fragmentation into multiple categories (academic years, docimological formats, and authors) resulted in small effect sizes that were insufficient to reach statistical significance.

### Future Work

To ensure the external validity of this methodology, our next step involves a large-scale, multicenter validation study spanning multiple medical specialties. Future research will focus on the external validation of these scores by correlating LLM-generated fragility signals with student psychometric data, such as bimodal response distributions or item discrimination indices. This will allow us to establish the predictive validity of our *a priori* tool against the *a posteriori* gold standard of student performance. Both qualitative and quantitative assessments of examinations will be implemented before and after the validation tool is deployed. Once validated, the system will be adopted for the *a priori* evaluation of faculty examination quality at the local level using LLMs. An online platform will provide easy access to this solution, facilitating its integration into academic routines.

### Conclusions

Our findings suggest a potential role that LLMs can play in medical education. We analyzed 264 exam questions across 3 academic years using four different LLMs. A standardized evaluation process was applied, combining automated scoring with 4 binary diagnostic tags to generate composite ambiguity and weighted quality scores. This methodology enabled a more standardized assessment of both individual questions and entire exam components. Overall, the models achieved performance levels comparable to students, with significantly higher scores on mPCC and IQS formats. IQS items exhibited the highest ambiguity scores, frequently associated with incoherence and low-performance tags. Ambiguity scores varied by year, with a predominance of moderate ambiguity (score 2) and occasional strong signals in IQS. Across all cohorts, IQS items were consistently represented, while the 2025 cohort included the full range of docimological formats. No significant differences in exam quality were observed based on the authors’ specialty or academic rank. Given the exploratory and monocentric nature of this pilot study, these results represent hypothesis-generating observations that necessitate further prospective validation.

LLMs act as intelligent assistants in a human-in-the-loop framework, flagging potential issues for expert review to improve the overall clarity and fairness of medical assessments. As these technologies evolve, more research is needed to explore their applications across disciplines and institutions.

## Supplementary material

10.2196/82702 Multimedia Appendix 1 Upset plot of tag combinations.

10.2196/82702 Multimedia Appendix 2 Comparison of quality scores between career levels, specialties, and docimological components.

## Abbreviations

AI: artificial intelligence
IQS: isolated question sequence
KFP: key feature problem
LLM: large language model
MCQ: multiple-choice question
mPCC: mini–progressive clinical cases
PCC: progressive clinical cases
SAQ: short-answer question
