# Supplementary figures and images for: Ambiguity Detection in Medical Exams via Large Language Models: Retrospective Cross-Sectional Pilot Study
Source: JMIR Med Educ. 2026 May 26;12:e82702. doi: 10.2196/82702 (PMC13211589; doi:10.2196/82702)

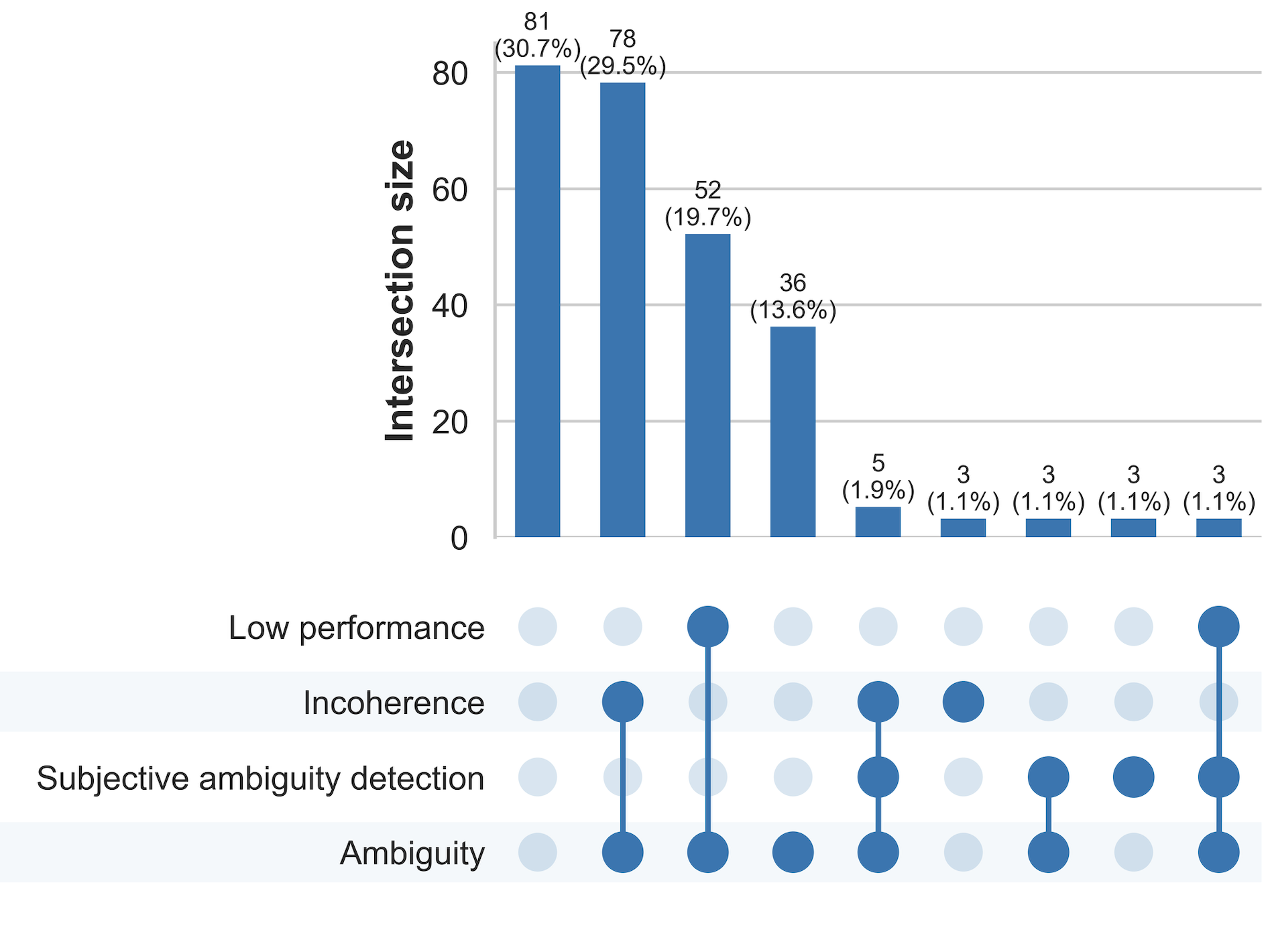

Supplement: Multimedia Appendix 1 [file mededu-v12-e82702-s001.png]
